# Nutritional factors regulating behavior and biological clocks

**DOI:** 10.20945/2359-3997000000410

**Published:** 2021-10-29

**Affiliations:** 1 Universidade Estadual de Campinas Centro de Pesquisa em Obesidade e Comorbidades Campinas SP Brasil Centro de Pesquisa em Obesidade e Comorbidades, Universidade Estadual de Campinas, Campinas, SP, Brasil

I was recently invited to write an editorial (1) about an excellent review (2) that put together a large amount of data showing the impact of nutrients on the regulation of gene expression and systemic inflammation. Over the last 50 years, changes in nutritional standards taking place in most regions of the world have reshaped human health parameters promoting an unprecedent increase in the prevalence of obesity and its comorbidities (3). As shown by Correa and cols. (2), components of the diet can regulate systemic metabolism by different means, such as activating cell receptors, modulating inflammation, promoting epigenetic modifications, and acting through microRNAs. Over the years, researchers have engaged most of their efforts to characterize the harmful and beneficial effects of nutrients on the most prevalent conditions associated with obesity, such as diabetes and cardiovascular diseases. However, there are several other health conditions that can be deteriorated in obesity. In this issue of the *Archives of Endocrinology and Metabolism*, two experimental studies provide advance in the mechanistic understanding of how dietary factors impact on anxiety-like behavior (4) and circadian clocks (5).

In humans, there is evidence supporting the association between obesity and anxiety/depression. A meta-analysis evaluating 16 studies reported an odds ratio of 1.4, indicating a moderate level of positive association between both conditions (6). Inflammation is believed to play an important mechanistic role in this scenario as in both obesity (7) and anxiety/depression (8) there is inflammation in distinct areas of the brain. Currently, pediatric obesity is one of the most serious health problems in the world. A child that develops obesity early in life can present cardiovascular disease up to a decade earlier than baseline population (9). However, it is uncertain how the early development of obesity could affect behavior. With this question in mind, Lorena and cols. (4) evaluated an animal model fed on a high-fat diet from early life. They showed that markers of inflammation were significantly increased in the prefrontal cortex of obese rats and this was accompanied by behavioral changes compatible with the anxiety/depression phenotype. Because of the existence of confounding factors, the determination of behavioral phenotypes in animal models is complex (10). To avoid biases, experimental approaches are optimized by the execution of several tests. In their model, Lorena and cols. (4) performed seven distinct tests to explore depression, anxiety and memory. In two tests, forced swimming test and novelty-suppressed feeding test, obese rats presented abnormalities, suggesting they have developed an anxiety/depression-like behavior. The study provides an elegant experimental evidence to support the association between obesity at an early age with anxiety and depression (11), showing that diet-induced inflammation in the prefrontal cortex could provide a mechanistic link between both conditions.

Key components of the body energy homeostasis apparatus are under the control of circadian clocks that synchronize caloric intake and energy expenditure with environmental cues (12). Clock genes expressed in neurons of the suprachiasmatic nucleus orchestrate daily cycles that impact on whole body homeostasis (12), and mutations of genes that play central roles in this system promote changes in feeding rhythms leading to obesity (12). Studies have shown that the suprachiasmatic clock connects with other organs of the body providing a tissue specific network that warrant whole body synchronization with the surrounding environment (13). Rodrigues and cols. (5) have evaluated the impact of restricted feeding on the expression of clock genes in three tissues that play important roles in whole body energy homeostasis: the liver, the brown adipose tissue, and the perigonadal adipose tissue. They showed that restricting feeding to daytime (which is non-physiological to rodents) promoted a shift in the expression of *Clock* and *Bmal1* in the liver, and to a lesser magnitude in the adipose tissue depots. This was accompanied by a shift in the pattern of expression of genes related to nutrient sensing, such as *Sirt1*, *Pgc1a*, *Pparg* and *Ucp2*. The study provides an interesting experimental proof-of-concept that changing feeding time, without restricting the amount of food is sufficient to modify clock genes in tissues involved in the regulation of systemic metabolism.

Taken together, the two studies add important experimental evidence to support a broad and complex interaction between nutritional factors and metabolic diseases. It has become clear that, not only the amount and quality of food, but also the time of the day food is consumed may impact on health. These concepts should provide the basis for a healthy nutritional education of the population.
